# High-brightness organic light-emitting diodes for optogenetic control of *Drosophila* locomotor behaviour

**DOI:** 10.1038/srep31117

**Published:** 2016-08-03

**Authors:** Andrew Morton, Caroline Murawski, Stefan R. Pulver, Malte C. Gather

**Affiliations:** 1 Organic Semiconductor Centre, SUPA, School of Physics and Astronomy, University of St Andrews, North Haugh, St Andrews KY16 9SS, United Kingdom; 2 Institut für Angewandte Photophysik, Technische Universität Dresden, George-Bähr-Str. 1, 01062 Dresden, Germany; 3 School of Psychology and Neuroscience, University of St Andrews, St Mary’s Quad, South Street, St Andrews KY16 9JP, United Kingdom

## Abstract

Organic light emitting diodes (OLEDs) are in widespread use in today’s mobile phones and are likely to drive the next generation of large area displays and solid-state lighting. Here we show steps towards their utility as a platform technology for biophotonics, by demonstrating devices capable of optically controlling behaviour in live animals. Using devices with a *pin* OLED architecture, sufficient illumination intensity (0.3 mW.mm^−2^) to activate channelrhodopsins (ChRs) *in vivo* was reliably achieved at low operating voltages (5 V). In *Drosophila melanogaster* third instar larvae expressing ChR2(H134R) in motor neurons, we found that pulsed illumination from blue and green OLEDs triggered robust and reversible contractions in animals. This response was temporally coupled to the timing of OLED illumination. With blue OLED illumination, the initial rate and overall size of the behavioural response was strongest. Green OLEDs achieved roughly 70% of the response observed with blue OLEDs. Orange OLEDs did not produce contractions in larvae, in agreement with the spectral response of ChR2(H134R). The device configuration presented here could be modified to accommodate other small model organisms, cell cultures or tissue slices and the ability of OLEDs to provide patterned illumination and spectral tuning can further broaden their utility in optogenetics experiments.

Optogenetics has emerged as a core technology in neuroscience to manipulate and record optically the activity of neurons^1^. The term is most widely used to define experiments in which the electrophysiological output of neurons expressing channelrhodopsins (ChRs) is modified in response to illumination with a specific wavelength of light^2^. ChRs are a class of light-sensitive ion channel proteins, originally identified for their essential role in phototaxis of single-cell green algae^3^,^4^. They became synonymous with optogenetics after ChR2 was successfully expressed in neurons in culture and in several model organisms, where it reliably drove neuronal spike-firing in response to illumination with blue light^5^,^6^,^7^,^8^,^9^.

ChRs mediate changes in neuronal excitability through light-induced photoisomerisation of an all-*trans* retinal molecule covalently anchored within the channel pore. The resultant changes in protein conformation cause transient channel opening and cation influx, leading to a depolarisation of neuronal transmembrane voltage that triggers action potential firing. Many ChR variants have emerged since ChR2 was originally deployed, with altered action spectra, ion selectivity, activation/inactivation kinetics and optical sensitivity among other properties^10^,^11^,^12^,^13^. This has resulted in an ever-expanding toolset of ChRs with which neurons can be activated, silenced or otherwise manipulated over wide-ranging timescales, with light of wavelengths from across the visible spectrum.

The methods for light delivery in optogenetics initially evolved somewhat more slowly. Conventionally, experiments on cell cultures or small model organisms are performed with filtered illumination from arc lamps, or with bulk high-power light-emitting diodes (LEDs) or lasers. Temporal modulation of optical excitation from such sources, to reproduce endogenous patterns of neural activity, can be achieved with motorised shutters, the driver electronics for LEDs, or external devices such as acousto-optic modulators for lasers. More recently, there have been considerable efforts to customise light delivery methods for optogenetics, in particular with respect to bio-implantable light sources^14^,^15^,^16^,^17^. There is also strong interest in spatial patterning of optical stimulation to restrict optical excitation to defined cells or to excite multiple sites independently. Examples from *in vitro* studies include modulation of laser illumination with digital micromirror devices^18^, or the projection of illumination from arrays of microscopic LEDs^19^. Significant technological advances have been motivated by the desire to achieve spatial patterning *in vivo*^16^,^20^. These extend from bio-implantable devices based on microscale inorganic LEDs coupled with recording electrodes^21^,^22^, to holographic techniques for distributing laser illumination for use alongside fluorescent imaging of neuronal activity^23^,^24^.

Organic light-emitting diodes (OLEDs) possess a number of properties that render them potentially very useful for optogenetics. Like conventional inorganic LEDs, they have μs or sub-μs response times, enabling frequency-modulated optical stimulation to be delivered with sub-microsecond temporal resolution. However, compared to their inorganic counterparts, they can be more readily fabricated on thin, flexible or even elastic plastic films^25^,^26^,^27^ which is of potential benefit for implantable optical-neural interfaces. OLEDs can also be highly efficient, even when emitting in the blue part of the visible spectrum^28^,^29^,^30^ and thus generate little heat during high brightness operation, ameliorating tissue heating that can be a concern with existing light delivery systems. OLEDs can also offer considerable versatility in their individual spectral tuning, which is determined by the choice of luminophore^31^,^32^ and can be tuned further by introducing optical micro-cavities 33,34. Furthermore, emitters with different colours can be layered or multiplexed to form white OLEDs, giving complete coverage of the visible spectrum from a single device^35^.

Previous reports proposed the possibility of using OLEDs for optogentics^36^ and we have very recently demonstrated switching of ChRs in non-neuronal systems using OLED micro-arrays^37^,^38^. However, to the best of our knowledge, OLEDs have not yet been used to modulate the activity of neuronal networks. A main constraint before now has been that their maximum optical power densities fell below those required for efficient ChR activation; most optogenetics experiments use illumination power densities in the range of 0.1–10 mW.mm^−2^ to elicit action potential firing optically. Here, we report a working implementation of OLEDs providing sufficient illumination intensity to achieve optical activation of ChRs *in vivo* for optogenetics. Due to the excellent electrical performance of the *pin* OLEDs used here, driving voltages of 5 V were sufficient to evoke robust optogenetic responses, making these light sources highly attractive for integration with standard electronics.

## Results and Discussion

In this study we used a transgenic line of *Drosophila* (OK371-GAL4/UAS-H134R-ChR2) in which larvae in their third instar stage of development (about 4–7 days after fertilisation) express ChR2(H134R) in motor neurons^39^. In these animals, upon ChR2(H134R) activation with blue light, robust firing of action potentials is evoked in motor neurons, which generates muscular contractions that temporarily immobilise larvae^39^. With this behavioural response as our readout, we used the *Drosophila* larvae to test if OLEDs provide sufficient luminance intensities for optical activation of neuronal circuits expressing ChRs and to identify OLEDs with suitable spectral profiles.

**Figure 1:**
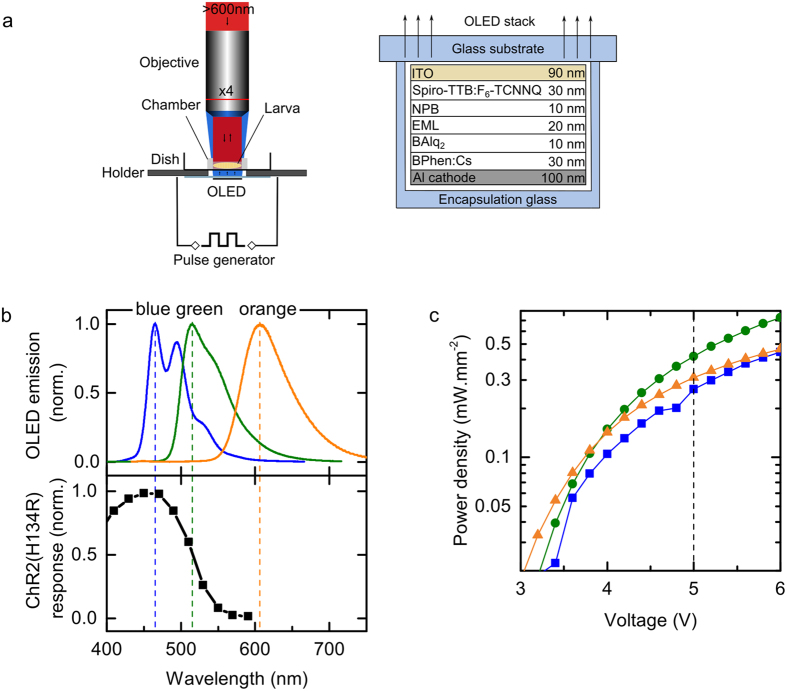
Optical setup, spectral characteristics of OLEDs compared to ChR2(H134R) and power density provided by OLEDs. (**a**) Left: OLEDs were contacted in a sample holder, mounted on an upright microscope and connected to a pulse generator. *Drosophila* larvae were mounted in a silicone chamber filled with sucrose solution, positioned above the OLED and imaged with red light. Right: Schematic illustration of *pin* OLED multi-layer stack architecture with abbreviation of material name and thickness of each layer. (**b**) Upper: Normalised emission spectra measured for the blue, green and orange OLED devices. Lower: ChR2(H134R) action spectrum^12^ with maxima from emission spectra for each OLED indicated by dashed lines. (**c**) Voltage-power density characteristics for blue (squares), green (circles) and orange (triangles) OLEDs.

In our experimental setup (Fig. 1a), an individual ChR2(H134R)-expressing larva was constrained in a silicone chamber containing sucrose solution and located above an OLED device. The animals were imaged with long-pass filtered light (>600 nm) to assess larval behaviour without background ChR activation. The OLEDs used here employ a *pin* structure where the active emissive layer (i) is sandwiched between doped hole (p) and electron (n) transport layers to reduce charge carrier injection barriers and voltage drop across the charge transport layers and thus achieve high brightness at low operation voltage^40^,^41^. For the blue-emitting OLEDs a fluorescent host-guest emitter system was employed, whereas phosphorescent emitters were used for the green- and the orange-emitting devices (see Methods for details of OLED fabrication and Fig. 1a for a schematic of the OLED stack).

The spectral profile of emission from all three OLEDs is shown in Fig. 1b. Our blue OLEDs peaked at 464 nm, matching well with the activation spectrum for ChR2(H134R), which is reported to peak around 450 nm^12^,^42^ (Fig. 1b, lower panel). Emission from green OLEDs (maximum: 515 nm) also overlapped the ChR2(H134R) action spectrum substantially (responses to 515 nm excitation are approximately 50% of the maximum^12^). Peak emission of the orange OLEDs was recorded at 606 nm and these devices showed negligible emission below 550 nm so were not expected to activate ChR2(H134R). Compared to conventional LED light sources, the OLED emission has a larger spectral bandwidth. However, as the activation spectra of ChR2(H134R) and most other ChR2 are even broader than the spectra provided by our OLEDs, we do not expect that this leads to a reduction in the efficiency of stimulation. If required, the bandwidth of the OLED emission can be reduced by employing optical microcavities^33^,^34^ or emitter systems with intrinsic narrow band emission^43^.

Figure 1c shows the voltage-power density characteristics for our blue, green and orange emitting OLEDs. For the following experiments, all OLEDs were driven with 5 V square wave pulses and at this voltage the different OLEDs provided comparable power densities (ranging between 0.25 and 0.4 mW.mm^−2^, Fig. 1c). We expect that a further substantial increase in power density can be achieved by further optimisation of device architecture, e.g. by reducing the voltage drop along the resistive indium tin oxide (ITO) connection lead and by fine-tuning of doping levels in the charge transport layers.

**Figure 2:**
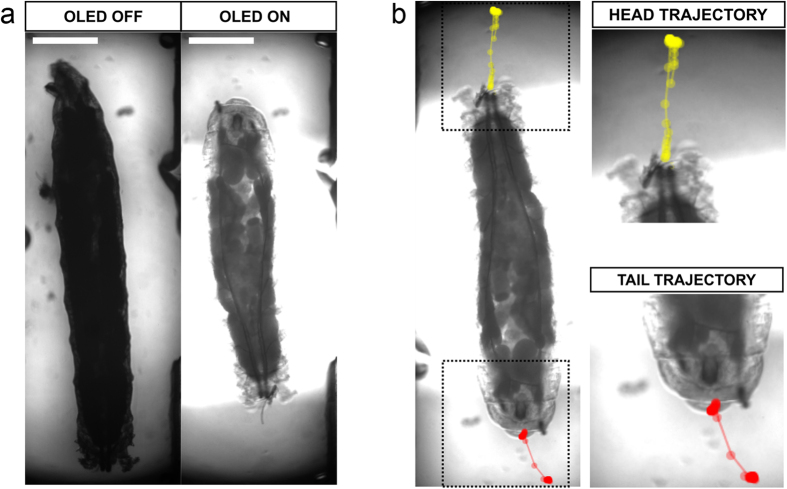
Imaging and analysis of *Drosophila* larval behaviour. (**a**) Example frames showing *Drosophila* larval morphology before (left) and during (right) blue OLED illumination. Scale bars: 0.5 mm. (**b**) Screen-capture images illustrating the analysis routine. Head and tail positions for each larva were tracked in each frame and head-tail distances calculated from these coordinates.

In resting conditions, larvae predominantly remained outstretched along their anterior-posterior axis, while exhibiting some feeding behaviour. Rapidly, upon blue OLED illumination, we observed a robust behavioural response in the form of a strong muscular contraction throughout the body plan of the larva, consistent with previous descriptions of responses to ChR2(H134R) activation in this model organism^39^. This manifested as a cessation of feeding behaviour, plus a rapid contraction that drew the head and tail of the larva closer together, followed by relaxation to the original elongated state after switching off the OLED (Fig. 2a; see also Supplementary Movie S1).

**Figure 3:**
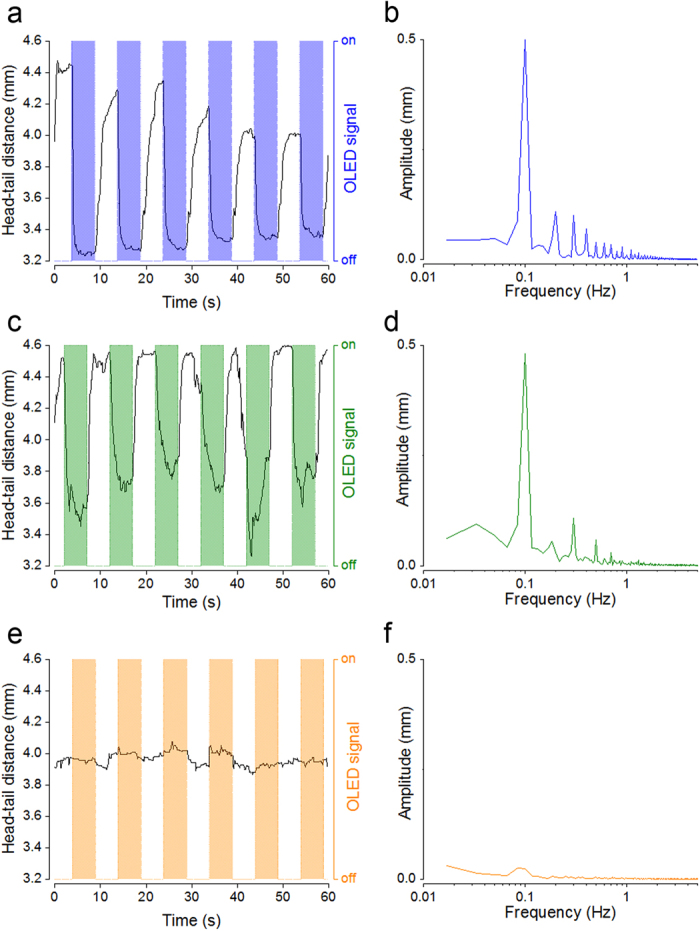
Movements in ChR2(H134R)-*Drosophila* larvae driven by optical stimulation with *pin* OLEDs. Repeated cycles of OLED illumination (5 s on −5 s off; 5 V square wave voltage pulse) were delivered from blue, green or orange OLEDs located beneath the larvae. Example head-tail distance traces (solid black lines) from larvae repeatedly stimulated with a blue (**a**), green (**c**) or orange (**e**) OLEDs. Shaded areas indicate when each OLED was on. (**b**,**d**,**f**) Corresponding Fourier transforms of each head-tail distance trace.

To measure the magnitude and kinetics of this behavioural response, we tracked head and tail positions of larvae exposed to alternating 5 s periods of OLED illumination and darkness. From these coordinates we then calculated a head-tail distance (Fig. 2b). During an imaging period of 60 s, sequential 5 s pulses of illumination from a blue OLED reliably triggered contractions in ChR2(H134R) larvae, appearing as shortenings in the measured head-tail distance (Fig. 3a). We investigated the temporal correlation between behaviour and timing of OLED illumination by performing a Fourier transform on the head-tail distance measurements. A dominant peak in amplitude in the transformed data was observed at 0.1 Hz (Fig. 3b), matching the frequency with which light pulses were delivered from the OLED (5 s illumination, followed by 5 s darkness). Thus, robust and repeatable optical activation of ChR2(H134R) *in vivo* was achieved with these blue OLEDs.

To examine the spectral selectivity of this behaviour and control for possible confounding factors such as changes in temperature or overall brightness, we replicated the experiment with green-emitting and orange-emitting OLEDs. Notably, when substituting with green-emitting OLEDs, we also observed a significant response of ChR2(H134R) larvae that was strongly temporally coupled to the OLED signal (Fig. 3c,d). In contrast, with an orange OLED, no discernible behavioural response could be attributed to optical stimulation (Fig. 3e,f).

**Figure 4:**
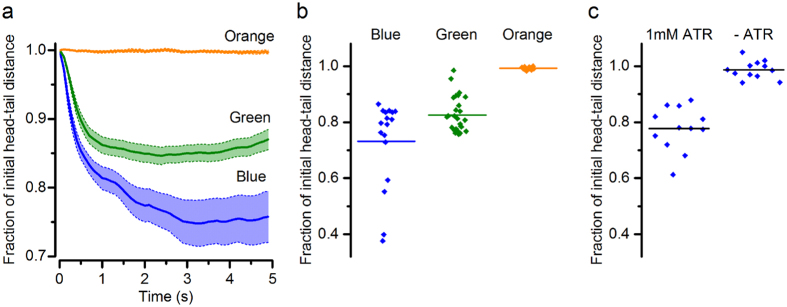
Analysis of contractions triggered in ChR2(H134R)-larvae by illumination with blue, green and orange OLEDs. (**a**) Larval contractions, shown as the mean fractional change in head-tail distance during 5 s periods of illumination delivered by blue, green and orange OLEDs. Error bands represent standard error of the mean. Blue OLED, n = 17 traces, from 4 larvae. Green OLED, n = 27 traces, from 6 larvae. Orange OLED, n = 11 traces, from 3 larvae. Data are expressed as a fraction of the initial length of each larva immediately prior to OLED illumination. (**b**) Minimum fractional head-tail distances reached during blue, green and orange OLED stimulation. Points are the minima from the individual traces from which the mean traces in (**a**) were calculated. Horizontal lines show the mean for each OLED. (**c**) Minimum fractional head-tail distance elicited with 5 s light pulses from a blue inorganic LED. Responses were compared for larvae raised on food supplemented with all-*trans*-retinal (1 mM ATR) versus larvae raised on food without retinal (-ATR). n = 12 responses from 3 larvae per condition; P < 0.05, two-tailed t-test.

To compare contractile responses across larvae and between experiments performed with differently-coloured OLEDs, head-tail distances for each 5 s period of OLED illumination were plotted after normalising to the initial length of each larva immediately before illumination (Fig. 4a). With blue and green OLEDs, head-tail movements started rapidly upon onset of illumination, detectable in the first frame of illumination (frame duration: 100 ms). We determined the initial rate of contraction during the first 0.5 s of illumination from the gradient of a linear fit to this initial period and scaled to the average starting length of larvae measured in this study (4.2 mm). We found that the rate of contraction was faster for blue OLEDs (1.3 mm.s^−1^) than green OLEDs (0.9 mm.s^−1^). Head-tail movements then gradually slowed, reaching minima by 2.4 s under green OLEDs and 3.3 s under blue OLEDs. Therefore, the initial rate and overall duration of head-tail movements initiated by green OLEDs were both roughly 70% of those induced by blue OLEDs.

The minima reached in the fractional head-tail distance traces were also compared for the different OLEDs (Fig. 4b). The largest overall behavioural responses in any conditions were observed in response to blue OLED illumination (mean fraction of initial head-tail distance ± standard error: 0.73 ± 0.04). Substantial responses, albeit slightly smaller overall, were also recorded from periods of green OLED illumination (0.83 ± 0.01). As expected, orange OLED illumination did not trigger detectable behavioural responses, with larvae remaining predominantly in their resting outstretched orientation. The minima reached for each OLED colour differed from each other significantly (ANOVA followed by Bonferroni test, α = 0.05).

Finally, for the purposes of comparison with an established light source for optogenetic stimulation, we measured the effectiveness of a blue inorganic LED at triggering behavioural responses in the same assay used to assess the OLEDs (Fig. 4c). Illumination from the blue LED (emission peak: 477 nm; 5 s on/5 s off) was relayed via a condenser lens from beneath the sample (power density on sample: 0.3 mW.mm^−2^) while imaging larvae as in the OLED experiments. The larvae showed behavioural responses much like those elicited by OLEDs, with contractions that were tightly coupled to the onset of optical stimulation from the blue LED and that were sustained for its entire duration (mean fraction of head-tail distance ± standard error: 0.78 ± 0.02). As an additional negative control, we also tested ChR2(H134R)-expressing larvae that had been cultured on growth medium not supplemented with all-*trans*-retinal (the essential cofactor for ChR activity). As expected, these animals did not exhibit contractions in response to the blue light stimulus (Fig. 4c).

In conclusion, we have demonstrated that OLEDs are capable of robust *in vivo* ChR activation in *Drosophila* larvae and developed a simple setup that could be readily adapted for other small model organisms, or for neuronal cultures or brain slices. Such a system will enable rapid prototyping of new devices with modified layouts and emitters, with a view to optimising further OLEDs for optogenetics.

In the future, by using thin-layer encapsulation, OLEDs may enable lens-free devices in which cells or tissue can be brought to within micron proximity of the emissive surface. Physical patterning of emissive pixels at microscopic resolutions can thereby allow single cells, and even subcellular compartments, to be addressed optically while mitigating the effects of divergence of light due to the close proximity between the cells and the light source. This has been recently implemented to control phototactic behaviour of the highly light sensitive single-cell green alga *Chlamydomonas reinhardtii*^37^ and the membrane potential of non-neuronal cells in culture^38^ but further improvements of the emission intensity are required to robustly trigger responses in neuronal systems. By combining high brightness OLEDs such as those used in this study, with further improvements in microscopic patterning, driver electronics, and thin film encapsulation (e.g. via combined organic/inorganic laminates), OLEDs may become a new method of choice for cellular-level optogenetic control of neuronal networks. OLEDs are also a candidate technology for bio-implantable devices for light delivery *in vivo.* A number of interesting properties of OLEDs could be harnessed for this purpose: devices can be (semi)transparent^44^,^45^, which may aid high-resolution *in vivo* imaging of neural activity; using stacked device architectures, OLEDs can emit several distinct spectral bands (e.g. blue and yellow light) that can be controlled separately^46^, which could be harnessed to address different optogenetic constructs at the same site; finally, devices can be mechanically flexible and could conceivably be integrated with analogous organic electronic devices for recording the activity of neurons^47^ to form highly conformable bi-directional bioimplants.

## Methods

### OLED fabrication

A series of three different types of OLEDs, emitting blue, green or orange light were produced. OLEDs were fabricated in an ultra-high vacuum chamber (Kurt J. Lesker Co.) at a base pressure of 10^−8^ mbar. The required organic materials were successively evaporated onto glass substrates coated with a 90 nm thick pre-structured indium tin oxide (ITO) anode. The thickness of each layer was monitored *in-situ* using quartz crystal monitors. The devices were composed of the following material stack (also see Fig. 1a): 30 nm 2,2’,7,7’-tetrakis(N,N’-di-p-methylphenylamino)-9,9’-spirobifluorene (Spiro-TTB) p-doped with 2,2’-(perfluoronaphthalene-2,6-diylidene)dimalononitrile (F_6_-TCNNQ) (2 wt %) as hole transport layer, 10 nm N,N’-di(naphtalene-1-yl)-N,N’-diphenylbenzidine (NPB) as electron blocking layer, 20 nm emission layer (EML, detailed below), 10 nm bis-(2-methyl-8-chinolinolato)-(4-phenyl-phenolato)-aluminium(III) (BAlq_2_) as hole blocking layer, and 30 nm cesium-doped 4,7-diphenyl-1,10-phenanthroline (BPhen) as n-doped electron transport layer, finished by a 100 nm thick aluminium cathode. For blue-emitting OLEDs, the EML consisted of the fluorescent emitter 2,5,8,11-tetra-tert-butylperylene (TBPe), which was doped with 1.5 wt% into the host 2-methyl-9,10-bis(naphthalen-2-yl)anthracene (MADN). For green OLEDs, the phosphorescent emitter fac-tris(2-phenylpyridine)iridium (Ir(ppy)_3_) was doped with 8 wt% into a double-EML of the hole-transporting 4,4’,4’’-tris(N-carbazolyl)-triphenylamine (TCTA) (8 nm) and the electron-transporting 2,2’,2’’-(1,3,5-benzinetriyl)tris(1-phenyl-1-H-benzimidazole) (TPBi) (12 nm). The orange EML comprised the phosphorescent emitter iridium(III)bis(2-methyldibenzo[f,h]quinoxaline)(acetylacetonate) (Ir(MDQ)_2_(acac)) doped with 10 wt% into an NPB host. All materials were purchased from commercial suppliers and purified further by vacuum gradient sublimation prior to use. All OLEDs were fabricated in one run using shadow masks and subsequently encapsulated under nitrogen atmosphere using glass lids and epoxy resin. Each substrate contained four identical OLEDs with an active area of 2.5 × 2.5 mm^2^.

### *Drosophila* imaging

OK371-GAL4/UAS-H134R-ChR2 flies were maintained at room temperature (20–22 °C) on solid cornmeal-based medium supplemented with 1 mM all-*trans*-retinal. Third-instar larvae were briefly washed in 5% (w/v) sucrose before being transferred to a small silicone chamber (Ibidi micro-Insert 4 well, Cat. No. 80409; the dividing wall between two adjacent wells was excised to create a well large enough to contain one larva) containing 5% (w/v) sucrose. This silicone chamber was positioned within a 35 mm cell culture dish and mounted on a custom-built sample holder for the OLEDs such that the larva was located above an OLED. The sample holder contained an array of pins positioned to contact the four ITO pads and the common metal cathode pad which are located at the perimeter of each OLED substrate. An OLED was mounted in the sample holder and this was connected to a pulse generator. This whole assembly was then mounted on an upright microscope (Nikon Eclipse Ni).

Sample illumination was from a fibre-coupled mercury light source (Nikon intensilight C-HGFI) attenuated (to <1 mW.mm^−2^) with neutral density (ND) filters and long pass filtered (>600 nm) to avoid non-specific activation of ChRs through imaging alone. Reflected light was collected with a 4X/0.13 NA air objective and slightly demagnified with an achromatic doublet lens (f = 50 mm) to collect the whole field of view on the sensor of the attached sCMOS camera (Andor Neo). Additional ND filters were placed in front of the camera, to prevent OLED emission saturating the detector. 60 s image sequences were acquired at 10 s^−1^, while externally modulating OLED emission with a pulse generator by applying square wave 5 V pulses of 5 s duration at 0.1 s^−1^. The optical power density provided by each OLED was measured by a calibrated power meter (Gentec-EO) with the photodiode (detector area: 10 mm Ø) placed in direct contact with the OLED. Emission spectra were recorded with a grating spectrograph coupled to a CCD detector (Andor).

For experiments with the blue inorganic LED, blue light pulses were projected onto the underside of the samples with a collimating lens. Spot size and driving current were adjusted to obtain an optical power density of 0.3 mW.mm^−2^. Responses to blue LED stimulation were compared between ChR2(H134R)-expressing larvae grown on media either supplemented or not supplemented with 1 mM all-*trans*-retinal.

### Analysis

Movies of *Drosophila* behaviour were imported as image sequences into the FIJI distribution of ImageJ. Using the plugin MtrackJ^48^ the position of the head and tail of each larva was manually recorded in each frame, then from these coordinates, a head-tail distance was calculated geometrically. Periods of OLED illumination were clearly apparent in the movies, so OLED illumination timings were reconstructed directly from the image sequences by placing a region of interest over the OLED and extracting an intensity value for each frame. Fourier transformation of head-tail distance traces and all numerical and statistical analyses were performed in Origin.

### Data availability

The research data supporting this publication can be accessed at http://dx.doi.org/10.17630/75928445-e77c-4f79-9b7f-d7d697ab7f29.

## Additional Information

**How to cite this article**: Morton, A. *et al*. High-brightness organic light-emitting diodes for optogenetic control of *Drosophila* locomotor behaviour. *Sci. Rep.* **6**, 31117; doi: 10.1038/srep31117 (2016).

## Supplementary Material

Supplementary Information

Supplementary Movie S1
