# Supplementary material for: High-brightness organic light-emitting diodes for optogenetic control of Drosophila locomotor behaviour
Source: Sci Rep. 2016 Aug 3;6:31117. doi: 10.1038/srep31117 (PMC4971487; doi:10.1038/srep31117)
Supplement: Supplementary Information [file srep31117-s1.pdf]

Supplementary Information

High-brightness organic light-emitting diodes for optogenetic control of *Drosophila* locomotor behaviour

Andrew Morton [1], Caroline Murawski [1,2], Stefan R. Pulver [3] & Malte C. Gather* [1,2]

1. Organic Semiconductor Centre, SUPA, School of Physics and Astronomy, University of St Andrews, North Haugh, St Andrews KY16 9SS, United Kingdom
2. Institut für Angewandte Photophysik, Technische Universität Dresden, George-Bähr-Str. 1, 01062 Dresden, Germany
3. School of Psychology and Neuroscience, University of St Andrews, St Mary's Quad, South Street, St Andrews KY16 9JP, United Kingdom

* Corresponding author, mcg6@st-andrews.ac.uk

Supplementary Movie S1: MortonGatherScientificReportsSupplementaryMovieS1.avi

An example video of an OK371-GAL4/UAS-H134R-ChR2 larva responding to optical stimulation supplied by a blue OLED. Field of view shown is 2 x 5 mm and playback speed is 30 fps.
